# Danuglipron Ameliorates Pressure Overload‐Induced Cardiac Remodelling Through the AMPK Pathway

**DOI:** 10.1111/jcmm.70488

**Published:** 2025-03-11

**Authors:** Pan Wang, Zhen Guo, Chun‐Yan Kong, Yu‐Lan Ma, Ming‐Yu Wang, Xin‐Ru Zhang, Zheng Yang

**Affiliations:** ^1^ Department of Cardiology Renmin Hospital of Wuhan University Wuhan P.R. China; ^2^ Hubei Key Laboratory of Metabolic and Chronic Diseases Wuhan P.R. China; ^3^ Department of Cardiology Zhongnan Hospital of Wuhan University Wuhan P.R. China; ^4^ Hubei Provincial Clinical Research Center for Cardiovascular Intervention Wuhan P.R. China; ^5^ Institute of Myocardial Injury and Repair Wuhan University Wuhan P.R. China

**Keywords:** autophagy, cardiac remodelling, Danuglipron (PF), GLP‐1 receptor agonist, HSP70

## Abstract

Cardiac remodelling, a pathological process induced by various cardiovascular diseases, remains a significant challenge in clinical practice. Here, we investigate the potential of Danuglipron (PF‐06882961, PF), a novel oral glucagon‐like peptide‐1 (GLP‐1) receptor agonist, in alleviating pressure overload (PO)‐induced cardiac hypertrophy and fibrosis. Using both in vivo and in vitro models, we demonstrate that PF treatment (1 mg/kg/day, orally for 8 weeks) significantly attenuates aortic banding‐induced cardiac dysfunction and pathological remodelling in mice. Mechanistically, we show that PF mitigates apoptotic responses and enhances autophagy by promoting AMPK phosphorylation and increasing HSP70 expression. Notably, the cardioprotective effects of PF are abolished in AMPKα2 knockout mice, with no observable increase in HSP70 levels. Our findings reveal a previously unrecognised role of PF in cardiac protection, mediated through the AMPKα‐HSP70 signalling pathway, and suggest its potential as a therapeutic strategy for PO‐induced cardiac remodelling.

## 1 Introduction

Heart failure (HF) is a complex clinical syndrome characterised by the heart's inability to meet the body's metabolic demands, resulting in significant morbidity and mortality worldwide [1]. Despite advances in pharmacological interventions, including β‐blockers, angiotensin receptor inhibitors, and neprilysin inhibitors, the progression of HF remains challenging to halt or reverse completely [2]. This underscores the urgent need for novel therapeutic strategies to effectively target the underlying pathophysiological mechanisms of HF.

Cardiac remodelling, a key pathological process in HF development, involves complex alterations in cardiomyocyte structure and function, extracellular matrix composition, and cellular signalling pathways [3]. Various factors, including mechanical stress, neurohormonal activation, and inflammatory mediators, contribute to this maladaptive process [3]. Understanding the molecular mechanisms governing cardiac remodelling is crucial for developing targeted therapies to prevent or reverse HF progression.

Recent studies have highlighted the potential of GLP‐1 receptor agonists in cardiovascular protection, extending beyond their established role in diabetes management [4]. These agents have demonstrated the ability to reduce oxidative stress, inflammation, and apoptosis while enhancing autophagy and mitophagy in cardiac tissue [4]. Our previous work with Semaglutide, a GLP‐1 receptor agonist, revealed its capacity to reverse pathological hypertrophy, fibrosis, and cardiac dysfunction in a PO‐model by modulating energy metabolism and inhibiting pro‐fibrotic signalling pathways [5]. Dulaglutide, another GLP‐1 receptor agonist, prevents diabetic HF and favourably affects myocardial metabolic remodelling by impeding mitochondria fragmentation [6]. These findings collectively underscore the potential of GLP‐1 receptor agonists in addressing various aspects of cardiac pathology. In this context, Danuglipron (PF‐06882961, PF), a novel second‐generation oral GLP‐1 receptor agonist, has emerged as a promising candidate. While current clinical studies primarily focus on its glucose‐lowering effects and favourable safety profile in type 2 diabetes management [7], its potential cardiovascular benefits, particularly in the context of cardiac remodelling, remain largely unexplored. This gap in research presents a compelling opportunity to investigate whether PF's cardioprotective effects mirror those of other GLP‐1 receptor agonists, potentially offering a new therapeutic avenue for cardiovascular diseases.

AMP‐activated protein kinase (AMPK) plays a pivotal role in cellular energy homeostasis and has been implicated in cardioprotection through its regulation of autophagy, mitochondrial function, and anti‐apoptotic pathways [8]. The heat shock response, primarily mediated by heat shock factor 1 and its downstream effectors such as heat shock protein 70 (HSP70), represents another crucial cellular defence mechanism against stress‐induced damage [9]. Emerging evidence suggests a complex interplay between AMPK activation and the heat shock response in cardiovascular protection. Recent studies have demonstrated that various pharmacological agents can modulate this interaction to exert cardioprotective effects. For instance, metformin has been shown to alleviate cardiac hypertrophy and cardiomyocyte apoptosis by reducing mitochondrial reactive oxygen species production and stimulating the nuclear translocation of heat shock factor 1 [10]. Similarly, canagliflozin, an SGLT‐2 inhibitor, exhibits cardioprotective properties through AMPK activation and subsequent regulation of autophagy in cardiomyocytes [8]. Furthermore, dapagliflozin has been reported to regulate HSP70 activity via promotion of AMPK phosphorylation [11]. These findings collectively suggest that modulation of HSP70 expression through AMPK phosphorylation may represent a promising strategy to mitigate PO‐induced cardiac remodelling. However, the precise mechanisms underlying this AMPK‐HSP70 axis in the context of cardiac remodelling, particularly under pathological conditions, remain to be fully elucidated. Further investigation into this signalling pathway could potentially unveil novel therapeutic targets for cardiac hypertrophy and HF.

In this study, we aimed to investigate the cardioprotective effects of PF in PO‐induced cardiac remodelling and to delineate the underlying molecular mechanisms. We hypothesised that PF might attenuate pathological cardiac remodelling through modulation of the AMPK–HSP70 signalling axis, thereby influencing key cellular processes such as autophagy and apoptosis. By employing both in vivo and in vitro models, including AMPK knockout mice, we sought to provide comprehensive insights into the potential of PF as a novel therapeutic strategy for cardiac remodelling and HF.

## 2 Materials and Methods

### 2.1 Reagents

PF (98% purity), (R)‐(−)‐phenylephrine hydrochloride (PE), dimethyl sulfoxide (DMSO), and dorsomorphin (Compound C; BML‐275) were purchased from MedChemExpress (USA). All other chemicals were of analytical grade and obtained from commercial sources.

### 2.2 Animals and Treatments

Adult male wild‐type C57BL/6 mice (8–10 weeks old, body weight: 23.5–27.5 g) were purchased from the Institute of Laboratory Animal Science, Chinese Academy of Medical Sciences (China). All the experimental procedures were approved by the Animal Care and Use Committee of Renmin Hospital of Wuhan University (approval No. WDRM20220803B) and followed the Guidelines for the Care and Use of Laboratory Animals published by the US National Institutes of Health. This study was also performed in accordance with the ARRIVE guidelines.

First, we acclimated the C57BL/6 mice to the laboratory environment for 1 week. Then, we established a PO‐induced cardiac hypertrophy model in C57BL/6 mice using aortic banding (AB) surgery. All mice were randomly divided into six groups, sham + vehicle (*n* = 10), sham + PF (*n* = 10), AB (*n* = 10), AB + low‐dose PF (*n* = 10), AB + medium‐dose PF (*n* = 10), and AB + high‐dose PF (*n* = 10) (Figure 3), and given PF (1, 3, 9 mg/kg/day) or normal saline via oral gavage for 8 weeks starting from 3 days post‐surgery. AMPKα2 knockout mice (AMPKα2^−/−^) were generated and bred in our laboratory, as described in our previous article [12]. In the next experimental stage, we randomly assigned the experimental mice to four groups: WT (*n* = 10), sham‐AMPKα2^−/−^ + PF (*n* = 10), AB‐AMPKα2^−/−^ + vehicle (*n* = 10), and AB‐AMPKα2^−/−^ + PF (*n* = 10) with intragastric administration of PF (1 mg/kg/day) or saline for 8 weeks, which was initiated 3 days after AB surgery (Figure 7). After 8 weeks of feeding, the surviving mice were randomly selected for echocardiography to assess their cardiac function. Then the body weight, heart weight, lung weight, and tibial length of each mouse were measured and recorded after euthanized by administering excessive sodium pentobarbital (200 mg/kg, i.p.).

The concentration of PF is based on the daily oral doses of 30, 100, and 300 mg twice a day in humans [13]. Oral administration of PF at doses of 30, 100, and 300 mg, adjusted for a standard body weight of 70 kg, corresponds to 0.43 mg/kg (equivalent to 30 mg), 1.43 mg/kg (equivalent to 100 mg), and 4.29 mg/kg (equivalent to 300 mg). Therefore, we administered the mice drug doses of 1, 3, and 9 mg/kg/day via gavage, or an equal volume of control vehicle solution, for 8 weeks for subsequent experiments.

### 2.3 Echocardiography

We assessed the systolic and diastolic function of the left ventricle in mice by using ultrasound echocardiography (Vevo 3100 system Visual Sonics) fitted with a 30 MHz transducer. First, 2.5% isoflurane was given to quickly anaesthetise the mice, and then, 1.0%–1.5% isoflurane was given to maintain the anaesthetic state of the mice so that the heart rate of the mice was maintained at 450–550 beats per minute. On this basis, left ventricular two‐dimensional M‐mode echocardiography images were recorded for five consecutive cardiac cycles, and left ventricular end‐diastolic diameter (LVEDd), fractional shortening (FS), and left ventricular ejection fraction (LVEF) were measured.

### 2.4 Cell Culture

75% alcohol sterilises the suckling Sprague–Dawley rat that has just been born for 1–2 days and quickly removes its heart, which was rinsed and minced into small pieces. Digest the ventricular myocardium of the neonatal rat hearts using 0.125% trypsin solution prepared with D‐Hanks solution for 15 min. This digestion process was repeated four times, after which 16 mL of Dulbecco's modified Eagle's medium/nutrient mixture F12 (DMEM/F12, Gibco, USA) containing 4 mL of fetal bovine serum (FBS, Gibco) was added to terminate the digestion. The neonatal rat cardiomyocytes (NRCMs) were isolated using differential adhesion techniques and cultured in DMEM/F12 medium containing 15% FBS, 5‐bromodeoxyuridine (0.1 mM), and penicillin/streptomycin for 48 h. Next, we assessed the cytotoxicity of PF on cardiomyocytes. Cells were treated for 24 h with various concentrations of PF (0, 0.05, 0.1, 0.3, 0.9, 1.2 μg/mL) or an equal volume of vehicle. Cell viability was then determined using the enhanced cell counting kit (CCK‐8, Beyotime Biotechnology, China) according to the manufacturer's protocol. Absorbance was measured at 450 nm using a Synergy HT Multi‐Detection Microplate Reader (BioTek Instruments, USA). After determining the optimal concentration, cardiomyocytes were treated with the same volume of PF and vehicle for 96 h to determine the optimal treatment duration by assessing cardiomyocyte activity. To evaluate the effect of PF on cardiomyocyte hypertrophy, NRCMs were treated with various concentrations of PF (0, 0.05, 0.1, 0.3, 0.9 μg/mL) and an equal volume of vehicle, with or without the addition of PE (100 μM) for 24–48 h.

### 2.5 Morphological Analysis

Hearts were fixed in 10% formalin, embedded in paraffin, and sectioned transversely to obtain 5 μm‐thick heart slices. Haematoxylin and eosin (H&E) staining was performed on the heart sections from each group to evaluate cardiomyocyte cross‐sectional area (CSA), and picrosirius red (PSR) staining was used to assess the degree of myocardial fibrosis. Quantitative analysis of heart section morphology and collagen deposition was conducted using a digital image analysis system (Image‐Pro Plus 6.0 software). For each group, the average CSA of 30 fields of view was calculated, with a minimum of 200 cardiomyocytes per group, and collagen volume was measured.

### 2.6 Immunofluorescence

Immunofluorescence was used to analyse the expression of α‐actinin in NRCMs to assess the cell surface of cardiomyocytes. NRCMs were washed three times with PBS, each for 5 min, followed by fixation with 4% paraformaldehyde at room temperature for 15 min. After five washes with PBS, each for 3 min, the cells were permeabilised with 0.2% Triton X‐100 for 15 min. The NRCMs were then stained overnight with a monoclonal anti‐α‐actinin antibody (#69758, CST, USA), followed by incubation with Alexa Fluor 568‐conjugated goat anti‐mouse IgG secondary antibody (Invitrogen, USA) in a 37°C incubator for 1 h. After additional PBS washes, the cells were stained with DAPI for nuclear staining and mounted. Fluorescence images were captured using a fluorescence microscope (Olympus DX51, Japan). Image‐Pro Plus 6.0 software was used to measure individual cardiomyocytes. At 200× magnification, 6–10 microscopic fields were randomly selected with 3–5 cells counted per field, totaling 30–50 cells per group.

### 2.7 RT‐PCR and Western Blot Analysis

To measure the mRNA expression of cardiac hypertrophy and fibrosis‐associated markers, the total RNA was extracted from NRCMs or mouse left ventricular cardiac tissues using TRIzol (Invitrogen, USA) on ice, and cDNA was synthesised using the Transcriptor First Strand cDNA Synthesis Kit (Roche, Switzerland). Quantitative real‐time PCR (RT‐PCR) analysis was performed using the LightCycler 480 SYBR Green 1 Master Mix (Roche) according to the manufacturer's instructions. The primers used for RT‐PCR in the experiments are listed in Table S1.

The steps for protein extraction and western blot were conducted as described in our previous study [5]. The primary antibodies used in the experiments are as follows: Bax (ER0907) and active‐caspase 3 (ET1602‐47) were purchased from Huabio (China), while Bcl‐2 (#4223), p62 (#5114), Atg5 (#2630), AMPKα (#2532), phospho‐AMPKα (#2531), HSP70 (#4872), and GAPDH (#3683) were purchased from CST. The protein expression level of GAPDH was used as an internal standard.

### 2.8 Blood Glucose Measurements and Glucose Tolerance Test

In the modelled mice, the blood glucose levels in both standard and surgery animals were determined using a kit reagent (Exactech blood glucose strip) according to the glucose oxidase method with glucose test strips (Roche) mentioned in our previous article [5]. Fasting blood glucose measurements were performed weekly, while the glucose tolerance test (GTT) was performed 1 week before collecting peripheral blood and hearts. Specifically, mice fasted overnight, and blood glucose levels were measured at five time points: 15, 30, 60, 90, and 120 min after intraperitoneal glucose injection.

### 2.9 Total Alanine Transaminase, Aspartate Transaminase, and Creatinine Measurements

Orbital venous blood was collected from experimental mice and centrifuged for plasma. Serum concentrations of the liver enzymes alanine aminotransferase (ALT), aspartate aminotransferase (AST), and renal function indicator creatinine were determined by an automated biochemical analyser (ADVIA 2400 Siemens Ltd., USA) [5].

### 2.10 Statistical Analysis

Quantitative data are presented as mean ± SD unless otherwise stated, and statistical analysis was performed using GraphPad Prism 9.5.0 software. Comparisons between the two groups were performed using Student's *t*‐test. One‐way analysis of variance (ANOVA), two‐way ANOVA, and Tukey's post hoc test were used to assess differences among multiple groups. Statistical significance was defined as *p* < 0.05.

## 3 Results

### 3.1 PF Enhances Cardiomyocyte Viability Under PE‐Induced Stress

**FIGURE 1:**
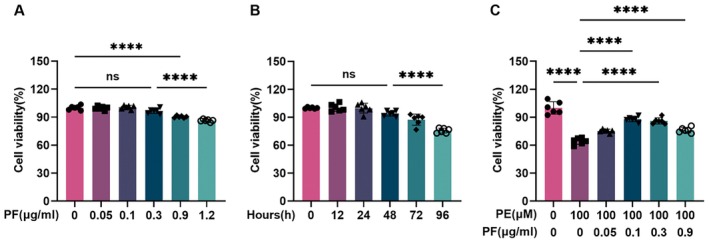
The effect of PF on the viability of NRCMs. (A) NRCMs were treated with different concentrations of PF (0, 0.05, 0.1, 0.3, 0.9, and 1.2 μg/mL), and cell viability was assessed to determine the dose‐dependent effects of PF on cardiomyocyte survival. (B) NRCMs were treated with 0.1 μg/mL PF for 0, 12, 24, 48, 72, and 96 h, followed by the assessment of cell viability at each time point. (C) In the presence of 100 μM PE, NRCMs were treated with varying concentrations of PF (0.05–0.9 μg/mL). Cell viability was measured to evaluate the protective effects of PF against PE‐induced stress. All data were expressed as mean ± SD. Statistical analysis was performed using one‐way ANOVA followed by Tukey's *post hoc* test. ns means no statistical significance, ****p < 0.0001.

To evaluate the potential effects of PF on cardiomyocyte survival, we assessed its impact on NRCM viability using the CCK‐8 assay. We first examined the dose‐dependent effects of PF on NRCMs. Cells were treated with varying concentrations of PF (0, 0.05, 0.1, 0.3, 0.9, and 1.2 μg/mL) for 24 h. As shown in Figure 1A, PF concentrations up to 0.9 μg/mL did not significantly affect cell viability compared to the control. However, at the highest concentration tested (1.2 μg/mL), a significant decrease in cell viability was observed. We next investigated the time‐dependent effects of PF on NRCM viability. NRCMs were treated with 0.1 μg/mL PF for various time periods (0, 12, 24, 48, 72, and 96 h). As illustrated in Figure 1B, cell viability remained stable up to 48 h of treatment. However, significant reductions in viability were observed at 72 and 96 h of continuous PF exposure. To assess the potential protective effects of PF against hypertrophic stress, we treated NRCMs with 100 μM PE alone or in combination with varying concentrations of PF (0, 0.05, 0.1, 0.3, and 0.9 μg/mL). As shown in Figure 1C, PE treatment alone significantly reduced cell viability compared to the control. Notably, co‐treatment with PF at concentrations of 0.1, 0.3, and 0.9 μg/mL significantly attenuated the PE‐induced decrease in cell viability, indicating a protective effect of PF against PE‐induced cellular stress.

Based on these results, we selected 0.1 μg/mL as the optimal PF concentration for subsequent experiments, as it demonstrated significant protective effects against PE‐induced stress without causing cytotoxicity within a 48‐h treatment period.

### 3.2 PF Has a Protective Effect on Myocardial Hypertrophy In Vitro

**FIGURE 2:**
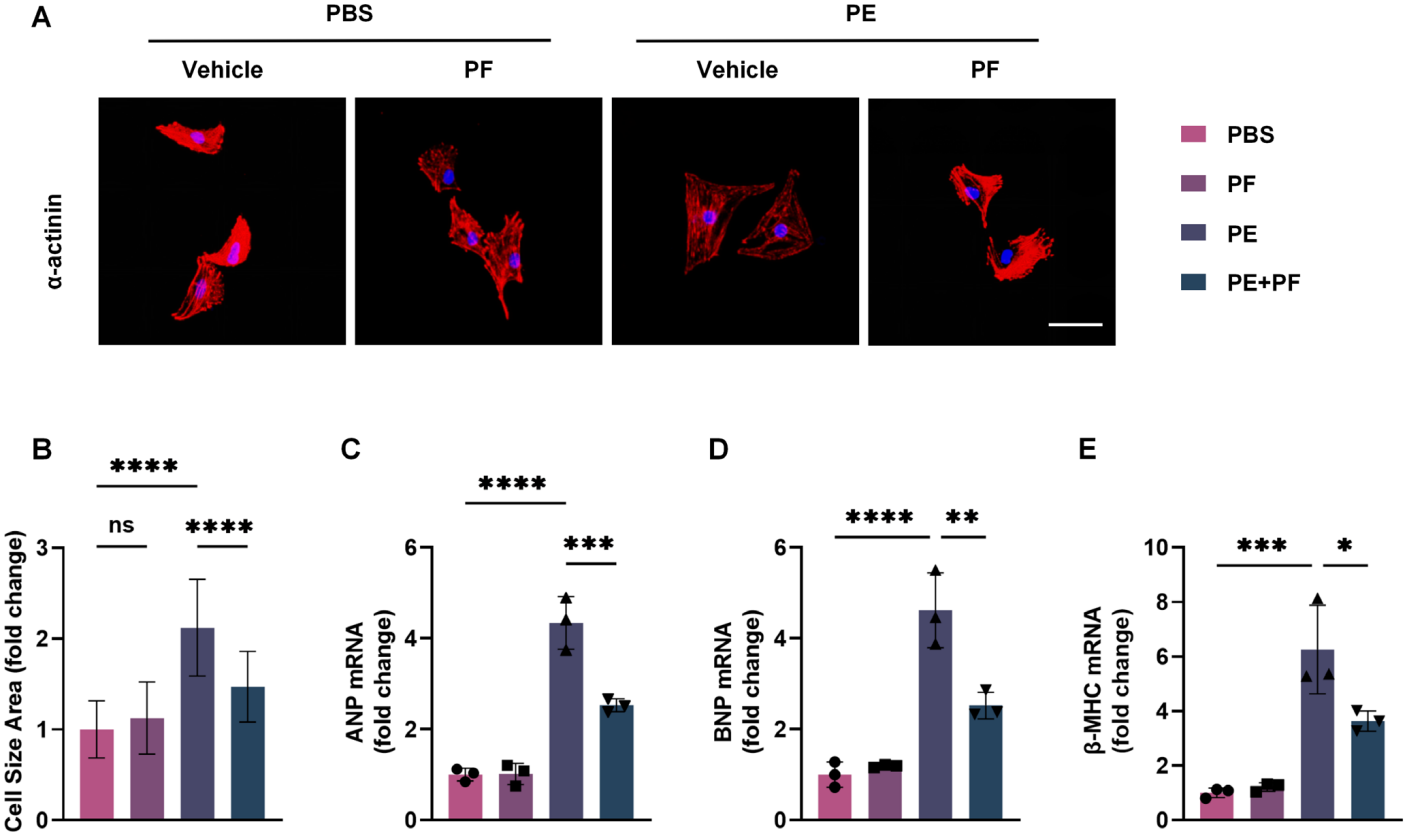
PF attenuates PE‐induced cardiomyocyte hypertrophy in vitro. (A) Representative immunofluorescence images of NRCMs stained for α‐actinin (red) under different treatment conditions: PBS (control), PF alone, PE alone, and PE + PF. Nuclei were counterstained with DAPI (blue). Scale bar: 50 μm. (B) Quantification of the NRCM cell surface area based on α‐actinin staining using ImageJ software. Data are presented as fold change relative to the PBS control group (*n* = 30–50 cells per group). (C–E) The expression of hypertrophy‐related markers (BNP, ANP, and β‐MHC) in NRCMs across different groups. Relative mRNA expression levels of hypertrophy‐related markers (ANP, BNP, and β‐MHC) in NRCMs under different treatment conditions, as determined by RT‐PCR. Data are normalised to GAPDH and expressed as fold change relative to the PBS control group (*n* = 3). All data are expressed as mean ± SD. Statistical analysis was performed using one‐way ANOVA followed by *post hoc* Tukey's test. ns means no statistical significance, *p < 0.05, **p < 0.01, ***p < 0.001, ****p < 0.0001.

To investigate the potential protective effect of PF against PE‐induced cardiomyocyte hypertrophy, we established an in vitro model using NRCMs. Cells were treated with 100 μM PE for 24–48 h to induce hypertrophy, with or without co‐treatment with PF at the previously determined optimal concentration of 0.1 μg/mL. Immunofluorescence staining for α‐actinin was performed to visualise and quantify changes in cell morphology. As shown in Figure 2A, PE treatment resulted in a marked increase in cardiomyocyte size compared to the control group. Notably, co‐treatment with PF significantly attenuated this PE‐induced enlargement. Quantitative analysis of the cell surface area (Figure 2B) revealed that PE treatment led to a 2.1‐fold increase in cell size compared to control, while PF co‐treatment reduced this increase to 1.5‐fold. To further confirm the anti‐hypertrophic effect of PF at the molecular level, we examined the mRNA expression of established hypertrophic markers: atrial natriuretic peptide (ANP), brain natriuretic peptide (BNP), and β‐myosin heavy chain (β‐MHC). RT‐PCR analysis (Figure 2C–E) demonstrated that PE treatment significantly upregulated the expression of all three markers. Importantly, PF co‐treatment significantly reduced the PE‐induced expression of these markers.

Collectively, these results provide strong evidence that PF effectively attenuates PE‐induced cardiomyocyte hypertrophy in vitro, as demonstrated by both morphological and molecular analyses.

### 3.3 PF Alleviates AB‐Induced Cardiac Dysfunction in Mice

**FIGURE 3:**
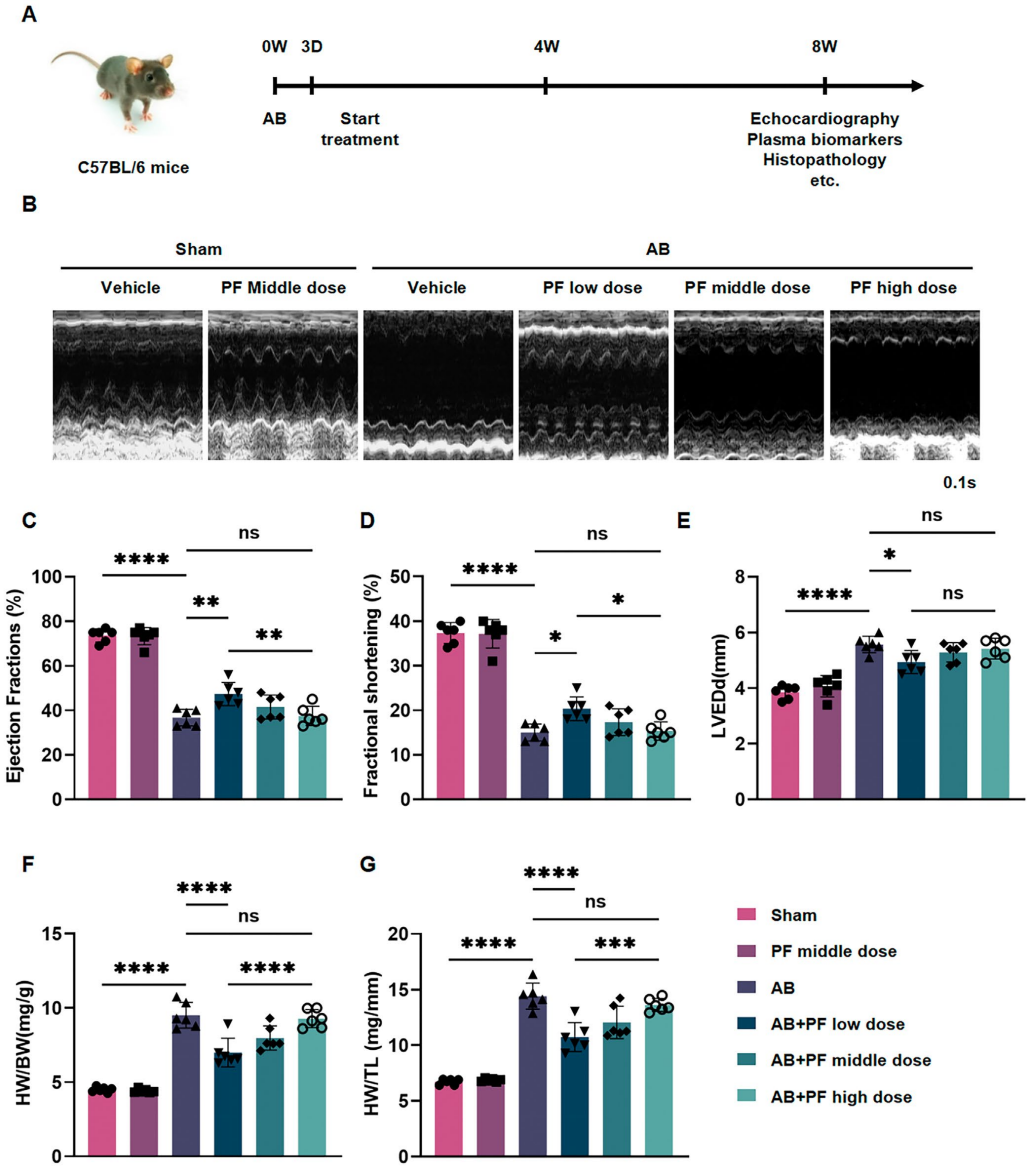
PF alleviates AB‐induced cardiac dysfunction in mice. (A) Schematic diagram of the experimental timeline. C57BL/6 mice underwent aortic banding (AB) surgery on day 0 (D0), followed by PF treatment initiation on day 3 (D3). Echocardiography, plasma biomarker analysis, and histopathological examinations were performed on 8 weeks. (B) Representative M‐mode echocardiographic images of left ventricular function in different treatment groups. (C–E) Quantitative analysis of echocardiographic parameters: ejection fraction (EF, %), FS (%), and LVEDd (mm). (F, G) Gravimetric analysis showing the HW/BW (mg/g) and HW/TL (mg/mm) (*n* ≥ 6). ns means no statistical significance, *p < 0.05, **p < 0.01, ***p < 0.001, ****p < 0.0001.

To investigate the effects of PF on left ventricular function in vivo, we established an AB‐induced cardiac hypertrophy model in C57BL/6 mice. As outlined in Figure 3A, mice underwent AB surgery followed by oral administration of PF or vehicle for 8 weeks. Echocardiographic assessment (Figure 3B) revealed that PF treatment alone did not significantly affect cardiac function in sham‐operated mice. However, AB surgery resulted in significant left ventricular dilation and dysfunction, as evidenced by decreased LVEF and FS, along with increased LVEDd (Figure 3C–E). Notably, PF treatment dose‐dependently improved these parameters in AB mice, with the low‐dose PF treatment showing the most pronounced effect. It significantly increased LVEF and FS, while reducing LVEDd compared to untreated AB mice (Figure 3C–E). Gravimetric analysis (Figure 3F,G) further supported the cardioprotective effects of PF. AB surgery significantly increased the ratios of heart weight to body weight (HW/BW) and heart weight to tibia length (HW/TL). PF treatment, particularly at the low dose, significantly attenuated these increases. Collectively, these results demonstrate that PF treatment, especially at a low dose, effectively alleviates AB‐induced cardiac dysfunction and hypertrophy in mice.

### 3.4 PF Attenuates AB‐Induced Cardiac Remodelling

**FIGURE 4:**
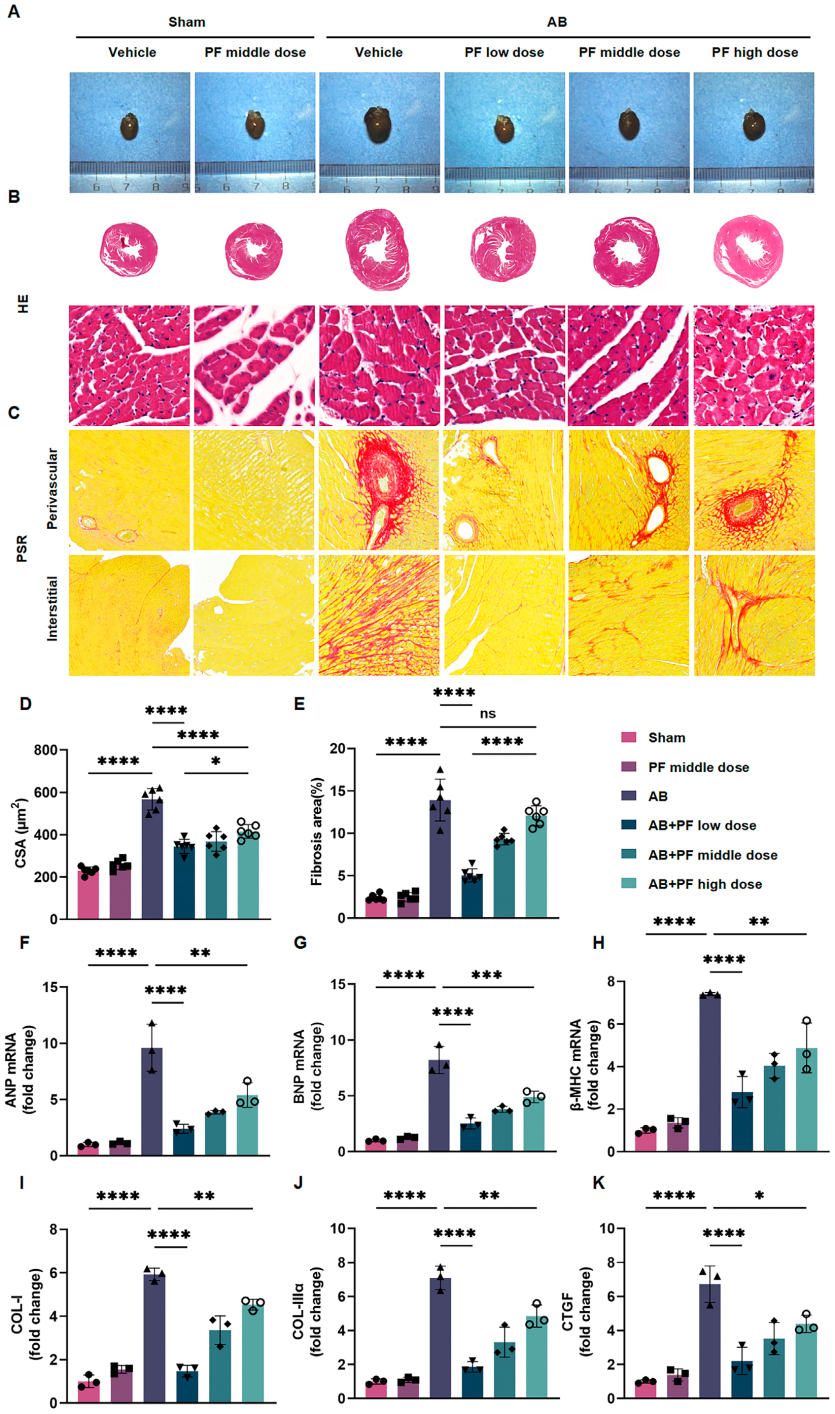
PF alleviated the progression of cardiac remodelling in mice in vivo. (A–C) Representative images of gross morphology of mouse hearts (A), (B) H&E‐stained cross‐sections of the hearts ((B) scale bar: 1000 and 50 μm), PSR‐stained sections (C) showing perivascular and interstitial fibrosis across different treatment groups. (D, E) Quantitative analysis of cardiomyocyte CSA ((D) μm^2^) and (E) fibrosis area (%) in the hearts of PF‐treated mice (*n* = 6). (F–H) Relative mRNA expression levels of hypertrophic markers (ANP, BNP, and β‐MHC) in mouse heart tissues, as determined by RT‐PCR (*n* = 3). (I–K) Relative mRNA expression levels of myocardial fibrosis markers (COL‐I, COL‐IIIα, and CTGF) in mouse heart tissues, as determined by RT‐PCR (*n* = 3). Data are presented as mean ± SD. ns means no statistical significance, *p < 0.05, **p < 0.01, ***p < 0.001, ****p < 0.0001.

To investigate the effects of PF on AB‐induced cardiac remodelling, we examined myocardial hypertrophy and fibrosis through gross morphological examination, histological analysis, and molecular marker expression. Gross examination (Figure 4A) and H&E staining (Figure 4B) revealed that hearts from AB mice were significantly enlarged, with hypertrophic cardiomyocytes compared to the sham group. PSR staining (Figure 4C) demonstrated markedly increased levels of both perivascular and interstitial fibrosis in AB mice. Notably, PF treatment dose‐dependently attenuated these AB‐induced changes, with the low‐dose PF showing the most pronounced effect.

Quantitative analysis confirmed that PF significantly reduced AB‐induced increases in CSA (Figure 4D) and fibrosis area (Figure 4E). The low‐dose PF treatment exhibited the most effective reduction in both parameters. At the molecular level, RT‐PCR analysis showed that PF treatment significantly downregulated the AB‐induced expression of hypertrophy markers (ANP, BNP, and β‐MHC; Figure 4F–H) and fibrosis markers (COL‐I, COL‐IIIα, and CTGF; Figure 4I–K). The suppression of these markers was most prominent in the low‐dose PF group.

To ensure that the observed cardioprotective effects were not due to systemic metabolic changes, we monitored various physiological parameters during the 8‐week PF treatment period. PF did not significantly affect body weight (Figure S1C), blood glucose levels (Figure S1A,B), or pancreatic islet function. Furthermore, liver function (ALT, AST) and kidney function (creatinine) remained unaltered in PF‐treated mice (Figure S1D–F).

These results collectively demonstrate that PF effectively attenuates AB‐induced cardiac remodelling, including both hypertrophy and fibrosis, without significantly impacting systemic metabolism or organ function.

### 3.5 PF Attenuates Cardiac Remodelling by Modulating Cardiomyocyte Apoptosis and Autophagy Pathways

**FIGURE 5:**
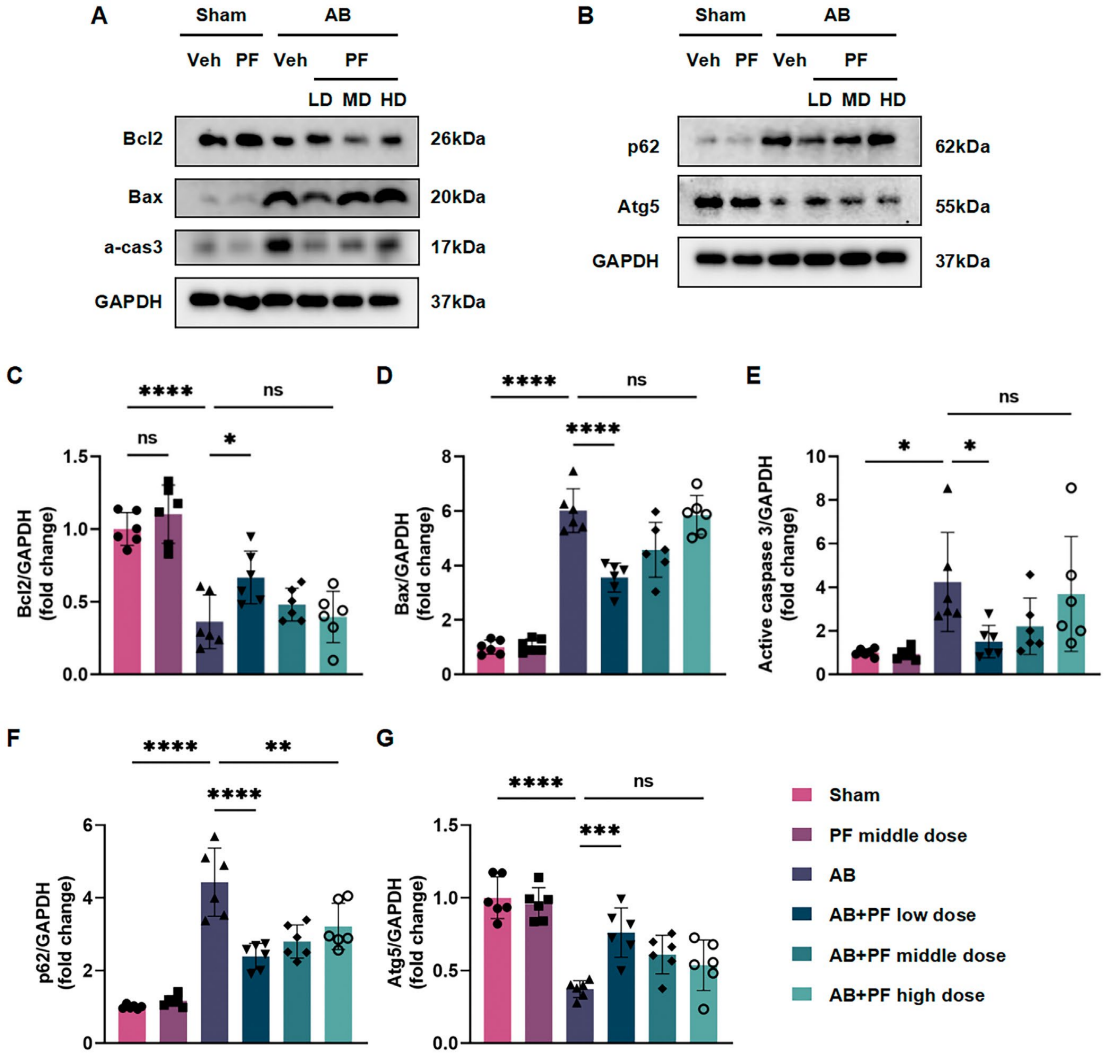
PF modulates apoptosis‐related proteins and autophagy markers in AB‐induced cardiac remodelling. (A) Representative western blot images showing the expression of apoptosis‐related proteins (Bcl‐2, Bax, and active caspase‐3) in heart tissues from C57BL/6 mice across different treatment groups. GAPDH serves as a loading control. (B) Representative western blot images showing the expression of autophagy markers (p62 and Atg5) in heart tissues from C57BL/6 mice across different treatment groups. GAPDH serves as a loading control. (C–E) Densitometric analysis of Bcl‐2, Bax, and active caspase‐3 protein levels normalised to GAPDH (*n* = 6). (F, G) Densitometric analysis of p62 and Atg5 protein levels normalised to GAPDH (*n* = 6). Statistical analysis was performed using one‐way ANOVA followed by *post hoc* Tukey test or unpaired *t*‐test. ns means no statistical significance, *p < 0.05, **p < 0.01, ***p < 0.001, ****p < 0.0001.

Apoptosis and autophagy are crucial processes in the development of AB‐induced cardiac hypertrophy [14]. To investigate whether PF attenuates cardiomyocyte hypertrophy by modulating these processes, we examined the expression of key apoptosis‐related proteins and autophagy markers in heart tissues from AB mice treated with different doses of PF. Western blot analysis revealed that AB surgery significantly altered the expression of apoptosis‐related proteins compared to the sham group (Figure 5A,C–E). Notably, PF treatment dose‐dependently increased the expression of the anti‐apoptotic protein Bcl‐2 while decreasing the levels of pro‐apoptotic proteins Bax and active caspase‐3. The low‐dose PF treatment showed the most pronounced effect in modulating these apoptosis‐related proteins.

Regarding autophagy, we focused on Atg5, a key protein involved in autophagosome formation, and p62, a protein that accumulates when autophagy is impaired [15]. In AB mice, we observed a significant increase in p62 expression and a decrease in Atg5 levels, indicating suppressed autophagy (Figure 5B,F,G). PF treatment, particularly at the low dose, significantly reversed these changes. It reduced p62 accumulation and increased Atg5 expression, suggesting a restoration of autophagic flux.

Interestingly, the effects of PF on both apoptosis‐related proteins and autophagy markers showed a non‐linear dose response, with the low dose generally exhibiting the most beneficial effects. These results collectively demonstrate that PF modulates both apoptosis and autophagy processes in AB‐induced cardiac remodelling. By suppressing pro‐apoptotic signalling and promoting autophagy, PF may contribute to the attenuation of pathological cardiac hypertrophy.

### 3.6 PF's Cardioprotective Effects Involve AMPKα Phosphorylation and HSP70 Expression

**FIGURE 6:**
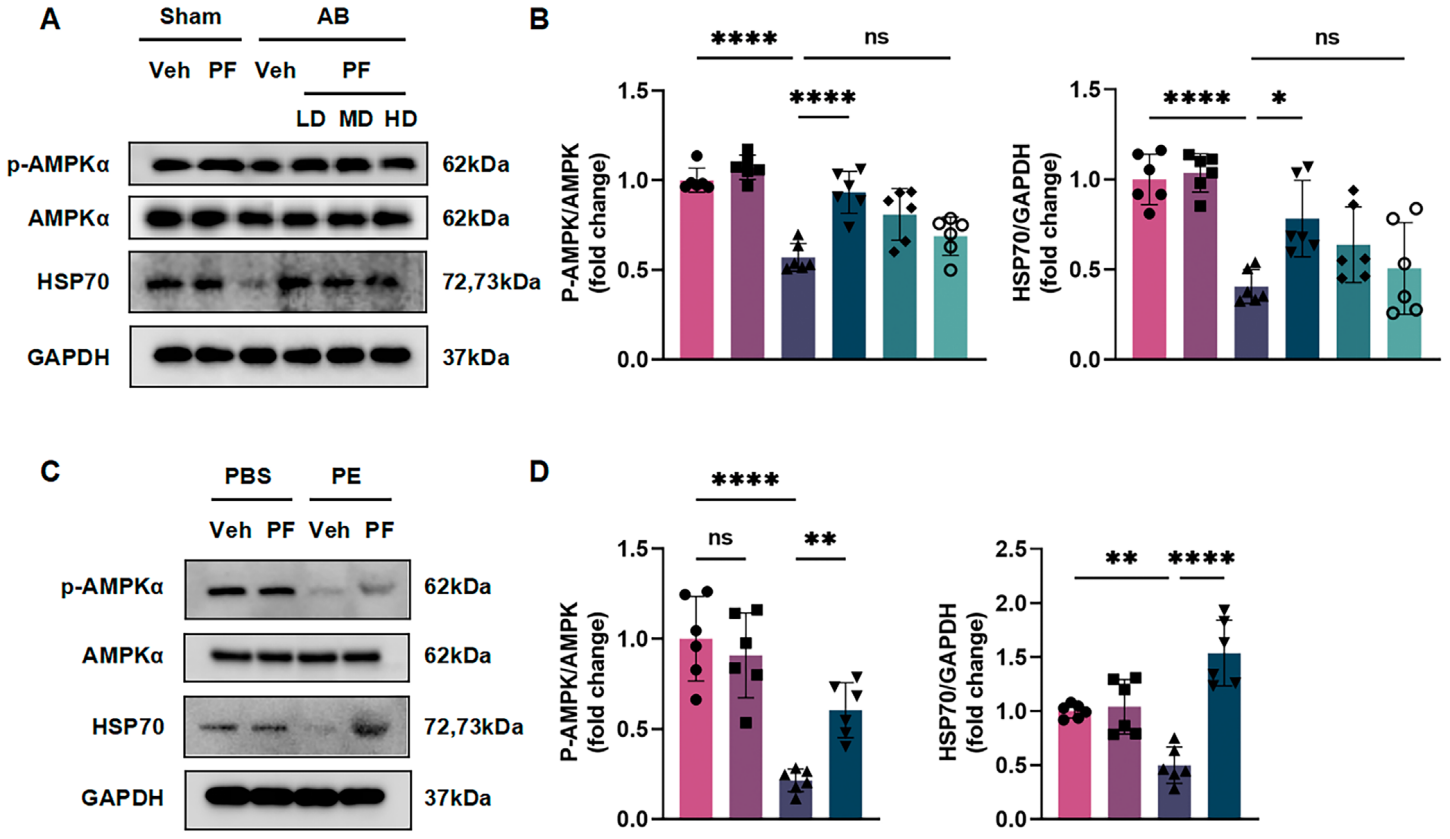
The cardioprotective effect of PF involves AMPKα and HSP70. (A) Representative western blot images showing the expression of p‐AMPKα, AMPKα, and HSP70 in heart tissues from C57BL/6 mice across different treatment groups. GAPDH serves as a loading control. (B) Densitometric analysis of the p‐AMPKα/AMPKα ratio and HSP70 protein levels normalised to GAPDH in mouse heart tissues (*n* = 6). (C) Representative western blot images showing the expression of p‐AMPKα, AMPKα, and HSP70 in NRCMs treated with PBS or PE, with or without PF. GAPDH serves as a loading control. (D) Densitometric analysis of the p‐AMPKα/AMPKα ratio and HSP70 protein levels normalised to GAPDH in NRCMs (*n* = 6). Blot analysis shows the expression of p‐AMPKα, AMPKα, and HSP70 in C57BL/6 mice hearts (*n* = 6). All data are presented as mean ± SD. Statistical analysis was performed using one‐way ANOVA followed by *post hoc* Tukey test or unpaired *t*‐test. ns means no statistical significance, *p < 0.05, **p < 0.01, ****p < 0.0001.

Our previous results demonstrated that PF attenuated AB‐induced cardiac remodelling. To elucidate the underlying molecular mechanisms, we investigated the involvement of AMPKα and HSP70, given their critical roles in regulating apoptosis and autophagy during PO‐induced cardiac hypertrophy [16]. We examined the phosphorylation of AMPKα (p‐AMPKα) and expression levels of HSP70 in both in vivo and in vitro models using western blot analysis. In C57BL/6 mice subjected to AB surgery, we observed a significant decrease in p‐AMPKα levels compared to sham‐operated controls (Figure 6A,B). Notably, PF treatment dose‐dependently increased p‐AMPKα levels in AB mice, with the low dose showing the most pronounced effect. These findings suggest that AMPKα phosphorylation may play a role in the pathological process of cardiac hypertrophy and PF's protective effects. Concurrently, we found that AB surgery significantly reduced HSP70 expression in heart tissues compared to the sham group (Figure 6A,B). PF treatment, particularly at low doses, significantly elevated HSP70 protein levels in AB mice.

To corroborate these findings, we conducted in vitro experiments using NRCMs treated with PE to induce hypertrophy. Consistent with our in vivo results, PE treatment decreased p‐AMPKα levels and HSP70 expression in NRCMs (Figure 6C,D). PF co‐treatment significantly reversed these changes, increasing both p‐AMPKα levels and HSP70 expression compared to PE‐treated cells.

These results collectively suggest that the cardioprotective effects of PF may be mediated, at least in part, through the modulation of AMPKα phosphorylation and HSP70 expression in both AB‐induced cardiac remodelling and PE‐induced cardiomyocyte hypertrophy.

### 3.7 AMPKα Phosphorylation Is Essential for PF‐Mediated Cardioprotection

**FIGURE 7:**
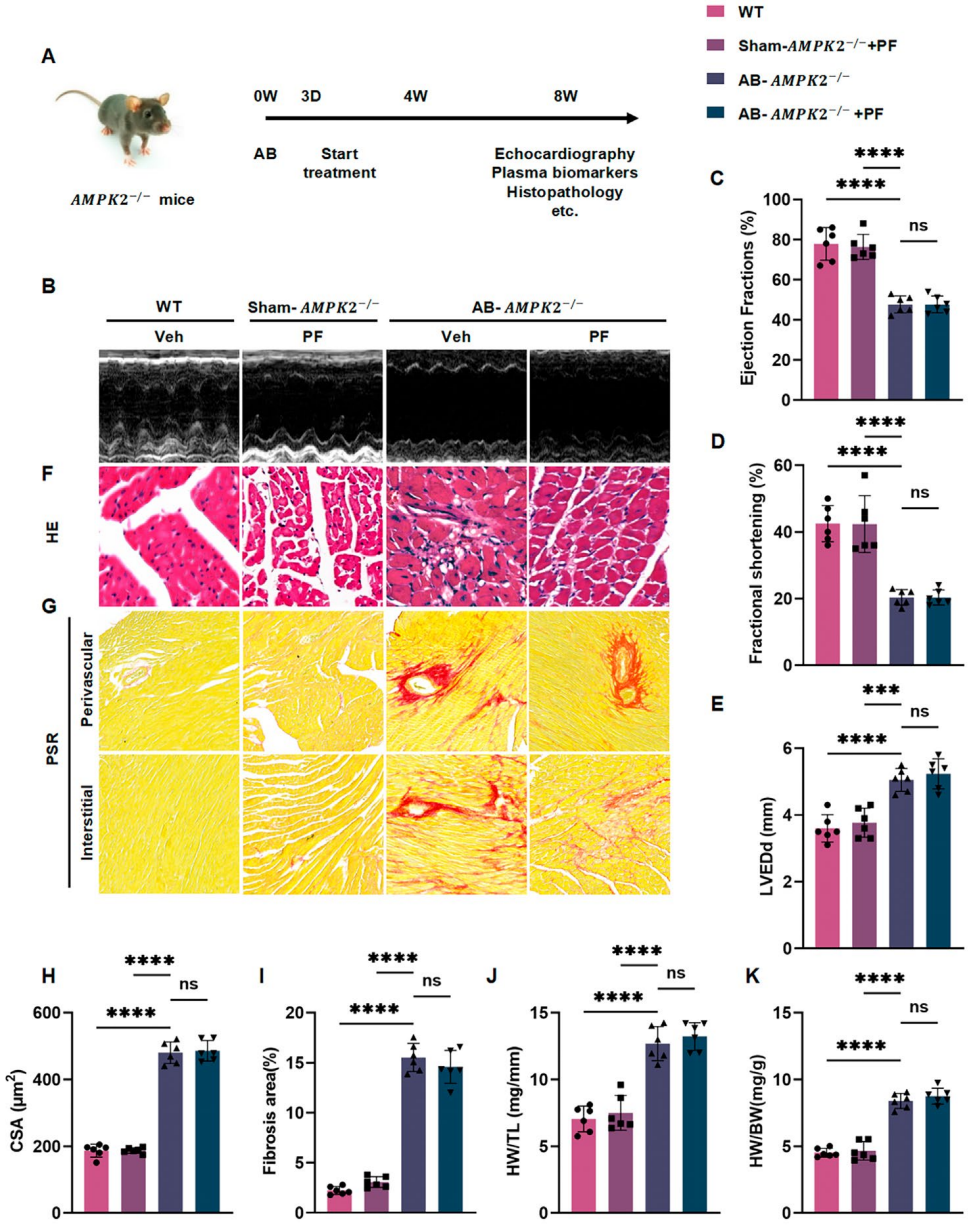
PF fails to attenuate AB‐induced cardiac remodelling in AMPKα2^−/−^ mice. (A) Diagram of animal experiment plan. (B) Representative M‐mode echocardiographic images of left ventricular function across different treatment groups. (C–E) Quantitative analysis of echocardiographic parameters: (C) EF (%), (D) FS (%), and (E) LVEDd (mm) (*n* ≥ 6 per group). (F) Representative images of H&E‐stained cardiac cross‐sections (scale bar: 50 μm). (G) Representative images of PSR‐stained cardiac sections showing perivascular (top) and interstitial (bottom) fibrosis (scale bar: 100 μm). (H, I) Quantitative analysis of cross‐sections and fibrosis in the hearts of AMPKα2^−/−^ mice treated with PF (*n* = 6). (J, K) Weight analysis of HW/BW and HW/TL (*n* ≥ 6). ns means no statistical significance, ****p < 0.0001.

To further verify the role of AMPKα phosphorylation in myocardial hypertrophy, we used AMPKα2^−/−^ mice as experimental subjects and established a myocardial hypertrophy model via AB surgery, followed by 8 weeks of PF treatment (Figure 7A). Cardiac protection by PF in AMPKα knockout mice was assessed through echocardiography, HE staining, and PSR staining (Figure 7B,F,G). Under physiological conditions, AMPKα2 knockout did not significantly alter the cardiac structure. However, following AB surgery, AMPKα2^−/−^ mice exhibited pronounced cardiac hypertrophy and fibrosis. Notably, PF treatment failed to attenuate these pathological changes in AMPKα2^−/−^ mice (Figure 7H,I). Echocardiographic analysis revealed that PF did not significantly improve LVEF or FS nor did it reduce LVEDd in AMPKα2^−/−^ AB mice (Figure 7C–E). Moreover, the HW/BW and HW/TL ratios remained elevated in PF‐treated AMPKα2^−/−^ AB mice compared to wild‐type and sham‐operated controls (Figure 7J,K).

**FIGURE 8:**
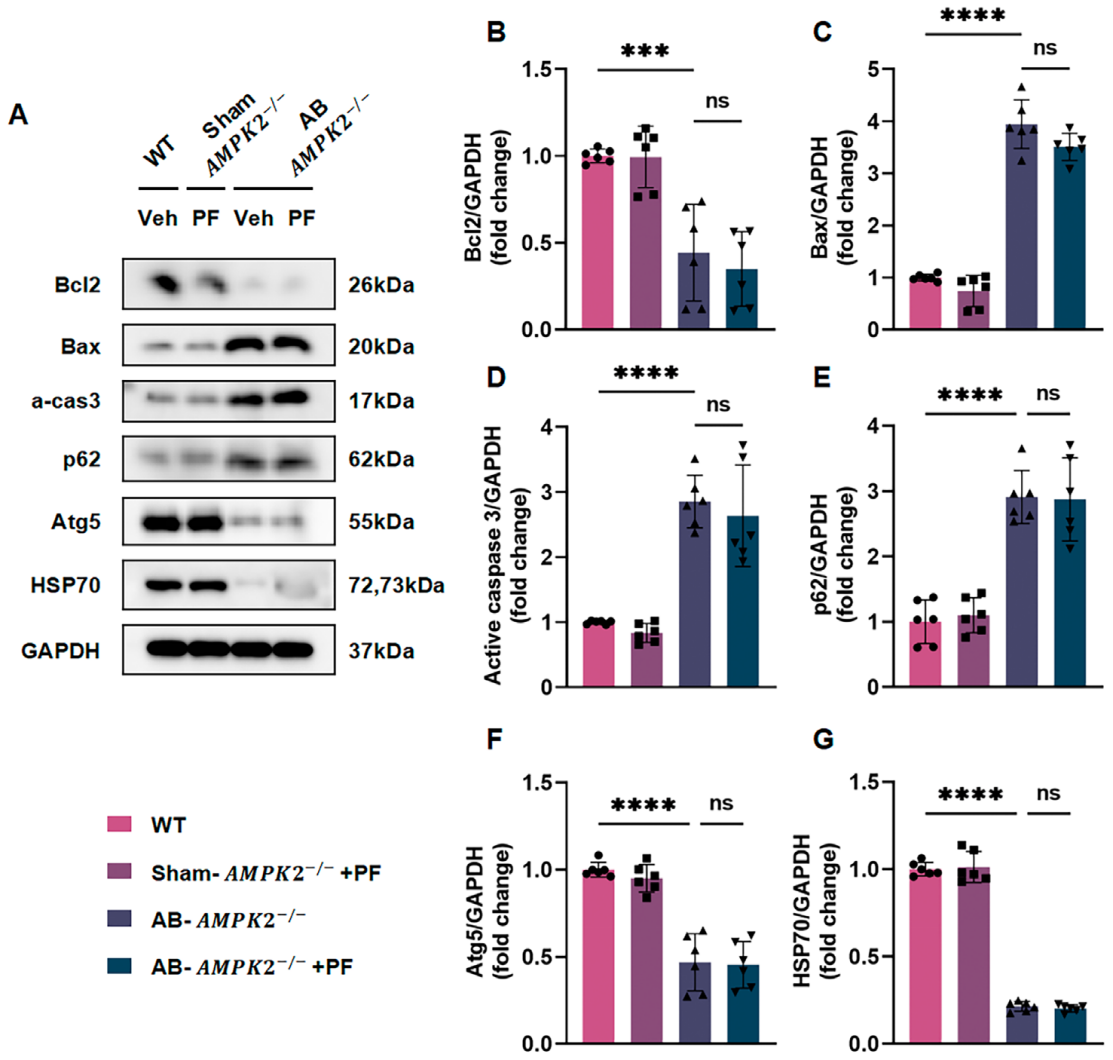
PF fails to modulate apoptosis and autophagy markers in AMPKα2^−/−^ mice with AB‐induced cardiac remodelling. (A) The expression of Bcl‐2, Bax, active‐caspase3, p62, Atg5, and HSP70 proteins in mouse hearts (*n* = 6). (B–G) Quantitative analysis of western blot data. All data are presented as mean ± SD. Statistical analysis was performed using one‐way ANOVA followed by *post hoc* Tukey test or unpaired *t*‐test. ns means no statistical significance, ***p < 0.001, ****p < 0.0001.

Molecular analysis of AMPKα2^−/−^ heart tissue using RT‐PCR and western blotting revealed that PF treatment did not significantly downregulate the expression of hypertrophy markers (ANP, BNP, and β‐MHC; Figure S2A) or fibrosis markers (COL‐I, COL‐IIIα, CTGF; Figure S2B). Furthermore, PF failed to modulate apoptosis‐related proteins in AMPKα2^−/−^ AB mice, as evidenced by decreased Bcl‐2 levels and increased Bax and active‐caspase‐3 levels (Figure 8A–D). Autophagy markers were also affected, with elevated p62 and reduced Atg5 expression (Figure 8A,E,F). Importantly, HSP70 expression remained significantly reduced in AMPKα2^−/−^ mice hearts, regardless of PF treatment (Figure 8G).

To further validate these findings, we employed an in vitro model using NRCMs treated with the AMPKα inhibitor Compound C. Consistent with our in vivo results, PF treatment of Compound C‐treated NRCMs failed to increase Bcl‐2, Atg5, and HSP70 levels or reduce Bax, active caspase‐3, and p62 levels (Figure S3A–G).

Collectively, these results strongly suggest that AMPKα phosphorylation is crucial for PF‐mediated cardioprotection, likely through the modulation of the AMPKα –HSP70 signalling pathway.

## 4 Discussion

**FIGURE 9:**
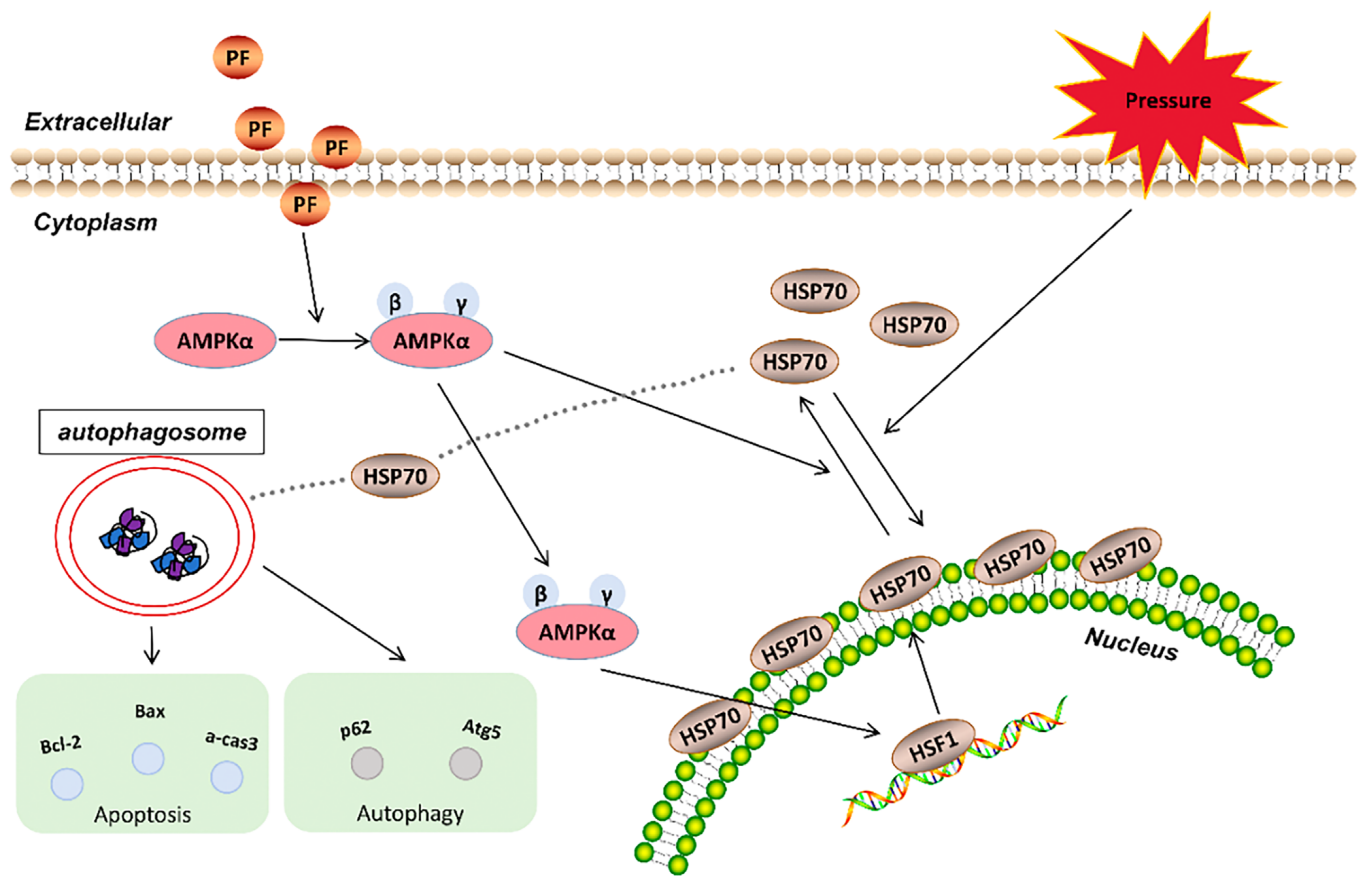
The underlying mechanism by which PF protects against cardiac remodelling. The treatment with PF, by promoting the AMPKα‐HSP70 signalling, activates autophagy response and alleviates apoptosis response during cardiac remodelling. HSF1, heat shock factor 1.

Cardiac remodelling, a critical process in the pathogenesis of HF, is often triggered by mechanical stress, including PO [17]. In this study, we utilised the AB model, a well‐established method to induce PO‐mediated cardiac remodelling in animal studies. Our findings demonstrate that PF, a novel oral GLP‐1 receptor agonist, exhibits significant cardioprotective effects in AB‐induced cardiac remodelling. Notably, we uncovered a pivotal role of the AMPK signalling pathway in mediating PF's protective effects. The efficacy of PF was substantially diminished in AMPK‐specific knockout models, underscoring the critical involvement of this pathway. Our results suggest that PF's cardioprotective mechanism involves the activation of AMPK phosphorylation, upregulation of HSP70 expression, and promotion of cardiomyocyte autophagy, collectively contributing to the inhibition of apoptosis during cardiac remodelling (Figure 9). These findings not only elucidate the molecular mechanisms underlying PF's cardioprotective effects but also highlight the potential of targeting the AMPK–HSP70 axis as a treatment for pathological cardiac hypertrophy and HF.

Our findings highlight the critical roles of autophagy and apoptosis in PF‐mediated cardioprotection during myocardial remodelling. Autophagy, a conserved cellular process involving lysosomal degradation of proteins and damaged organelles, plays a protective role in cardiomyocytes by mitigating oxidative stress and cellular damage [18, 19]. Conversely, excessive apoptosis, particularly in pathological conditions such as myocardial infarction and HF, can exacerbate cardiac remodelling and functional decline [20, 21]. In our study, PF treatment significantly modulated both these processes, enhancing autophagy markers (e.g., increased Atg5 expression) while reducing apoptotic indicators (e.g., decreased Bax/Bcl‐2 ratio). These observations align with previous studies demonstrating that cardioprotective agents can prevent cardiac remodelling by regulating autophagy and apoptosis‐related factors [22, 23, 24].

Our research extends previous findings by elucidating the specific involvement of the AMPK–HSP70 pathway in mediating PF's cardioprotective effects. AMPK promotes autophagy through various mechanisms, including the activation of UNC‐51‐like kinase 1 (ULK1), the recruitment of ATG proteins [25], and the modulation of mTOR phosphorylation [26]. PF treatment led to increased AMPK phosphorylation, associated with enhanced autophagy markers and reduced apoptotic indicators. This is consistent with the effects of AMPK activation on the AMPK‐FOXO3a signalling pathway, which upregulates autophagy‐related proteins like Beclin1 and LC3‐II/LC3‐I while modulating apoptosis‐related proteins such as Bcl‐2 and Bax [27]. Importantly, our study reveals a novel aspect by highlighting the role of HSP70 in conjunction with AMPK activation. While previous studies have demonstrated that GLP‐1 receptor agonists can regulate AMPKα expression and promote mitochondrial autophagy [28], our work is the first to elucidate the specific involvement of the AMPKα‐HSP70 axis in PF‐mediated cardioprotection. The upregulation of HSP70 by PF, likely through AMPK activation, may provide a mechanistic link between enhanced autophagy and reduced apoptosis, as HSP70 promotes protein quality control and cellular stress resistance [29]. This AMPK‐HSP70 axis appears to be a key mediator of PF's effects, potentially offering a more comprehensive protective mechanism compared to AMPK activation alone. Our findings align with and extend our previous research on AMPK activators in cardiac protection, such as Geniposide [30] and cytoplasmic P21 [31]. However, our work with PF is the first to elucidate the specific involvement of the AMPKα–HSP70 axis in GLP‐1 receptor agonist‐mediated cardioprotection, representing a novel direction in understanding the synergistic effects of these molecules in cardiac protection.

GLP‐1 receptor agonists have demonstrated diverse cardioprotective mechanisms, and our findings with PF reveal a novel pathway that expands this understanding. While previous studies have shown that agonists like semaglutide can improve cardiac function by optimising myocardial energy metabolism and promoting fatty acid oxidation [5] as well as downregulating NLRP3 inflammasome activation through enhanced mitochondrial autophagy [32]. Agonists like liraglutide protect against cardiac hypertrophy and fibrosis by activating the AMPK/mTOR/p70S6K pathway and ATP‐sensitive potassium channels [33, 34, 35]. Geniposide reverses cardiac dysfunction via both AMPKα‐ and Sirt1‐dependent mechanisms [30]. Our research suggests that PF operates through a distinct mechanism. PF's cardioprotective effects appear to be independent of systemic metabolic changes. We observed no significant alterations in body weight or blood glucose levels in PF‐treated groups, contrasting with the glucose‐dependent mechanisms of some other GLP‐1 receptor agonists. Instead, our study uniquely elucidates PF's molecular mechanism in myocardial protection through the AMPK‐HSP70 pathway. This was evidenced by the abolishment of PF's protective effects in AMPK‐specific knockout mice, underscoring AMPK's pivotal role and highlighting the novelty of the AMPK‐HSP70 axis in GLP‐1 receptor agonist‐mediated cardioprotection. Our findings thus not only expand the understanding of GLP‐1 receptor agonists' cardiac benefits beyond metabolic regulation but also suggest the potential for more direct and possibly more effective cardioprotective strategies in the treatment of heart failure and related conditions.

In this comprehensive evaluation of PF's impact on cardiac remodelling, we employed a dose–response approach, administering 1, 3, or 9 mg/kg/day of PF. Interestingly, we observed that cardiac remodelling showed less remission at higher PF concentrations than at lower doses. This non‐linear dose–response relationship warrants further investigation, as it may have important implications for optimal therapeutic dosing. The underlying mechanisms for this phenomenon, possibly involving receptor desensitisation or activation of compensatory pathways at higher doses, remain to be elucidated. Our study has several limitations that should be addressed in future research. First, the use of only male mice limits the generalizability of our findings across genders, emphasising the need for gender‐inclusive studies to fully ascertain PF's therapeutic potential. Second, while our research focused on the AMPK‐HSP70 pathway, other mechanisms, such as effects on inflammation or oxidative stress, may also contribute to PF's cardioprotective actions and deserve further exploration. It is noteworthy that PF exhibits species‐specific effects on glucose metabolism. Studies have shown hypoglycemic effects in primates but not in rodents [13], consistent with our observations. This highlights the complexity of translating our findings to human physiology, where the interplay between cardiac protection, metabolism, and blood sugar regulation may differ. Additionally, potential side effects of PF, primarily gastrointestinal reactions, need careful consideration in clinical applications. Despite these limitations, our elucidation of the AMPK‐HSP70 pathway in PF‐mediated cardioprotection provides a solid foundation for future research. Given the significant protective effects of GLP‐1 receptor agonists in cardiovascular diseases, future studies should focus on further characterising PF's mechanisms of action, including its effects on inflammation and oxidative stress. Moreover, clinical trials are needed to assess the efficacy and safety of PF in humans, with particular attention to optimising dosing regimens and mitigating potential side effects.

## 5 Conclusions

In conclusion, our study reveals the promising potential of PF in cardiovascular protection, particularly in mitigating PO‐induced myocardial hypertrophy through the novel AMPKα‐HSP70 signalling pathway. This mechanism extends the therapeutic applications of GLP‐1 receptor agonists beyond diabetes and obesity treatment, potentially independent of systemic glucose regulation. As an oral therapy, PF offers advantages in patient adherence and may revolutionise the treatment of metabolic and cardiovascular diseases. However, the observed non‐linear dose–response relationship warrants careful consideration in clinical applications. Future research should focus on further characterising the AMPKα‐HSP70 pathway, investigating PF's effects on inflammation and oxidative stress, and conducting gender‐inclusive studies and clinical trials. These efforts will be crucial in optimising PF's therapeutic potential and fully realising its benefits across diverse medical contexts, potentially leading to more targeted and effective therapies for cardiac diseases.

## Author Contributions

**Pan Wang:** conceptualization (lead), data curation (lead), formal analysis (lead), investigation (lead), methodology (lead), resources (lead), supervision (lead), validation (lead), visualization (lead), writing – original draft (lead). **Zhen Guo:** conceptualization (lead), data curation (lead), formal analysis (lead), investigation (lead), methodology (lead), resources (lead), supervision (lead), validation (lead), visualization (lead), writing – original draft (lead). **Chun‐Yan Kong:** formal analysis (equal), investigation (equal), validation (equal). **Yu‐Lan Ma:** formal analysis (equal), investigation (equal), visualization (equal). **Ming‐Yu Wang:** formal analysis (equal), investigation (equal), validation (equal). **Xin‐Ru Zhang:** formal analysis (equal), investigation (equal), validation (equal). **Zheng Yang:** conceptualization (equal), funding acquisition (equal), project administration (equal), resources (equal), supervision (equal), writing – review and editing (equal).

## Conflicts of Interest

The authors declare no conflicts of interest.

## Supporting information


Figures S1–S3

Table S1

## Data Availability

The data that support the findings of this study are available from the corresponding author upon reasonable request.
