# Supplementary material for: Danuglipron Ameliorates Pressure Overload‐Induced Cardiac Remodelling Through the AMPK Pathway
Source: J Cell Mol Med. 2025 Mar 11;29(5):e70488. doi: 10.1111/jcmm.70488 (PMC11897056; doi:10.1111/jcmm.70488)
Supplement: Supplementary file 1 — Figures S1–S3 [file JCMM-29-e70488-s001.pdf]

Supplemental material of Danuglipron ameliorates pressure overload-induced cardiac remodeling through the AMPK pathway.

Author Pan Wang[1,2#], Zhen Guo[3,4#], Chun-Yan Kong[1,2], Yu-Lan Ma[1,2], Ming-Yu Wang[1,2], Xin-Ru Zhang[1,2], Zheng Yang[1,2*]

[*]**Corresponding author:** Zheng Yang, Email: dr_yangzheng@whu.edu.cn

Author's affiliation

[1] Department of Cardiology, Renmin Hospital of Wuhan University, Wuhan 430060, RP China

[2] Hubei Key Laboratory of Metabolic and Chronic Diseases, Wuhan 430060, RP China

[3] Department of Cardiology, Zhongnan Hospital of Wuhan University, Wuhan, 430062, China;

[4] Institute of Myocardial Injury and Repair, Wuhan University, Wuhan, 430062, China.

[#] These authors contributed equally: Pan Wang, Zhen Guo.

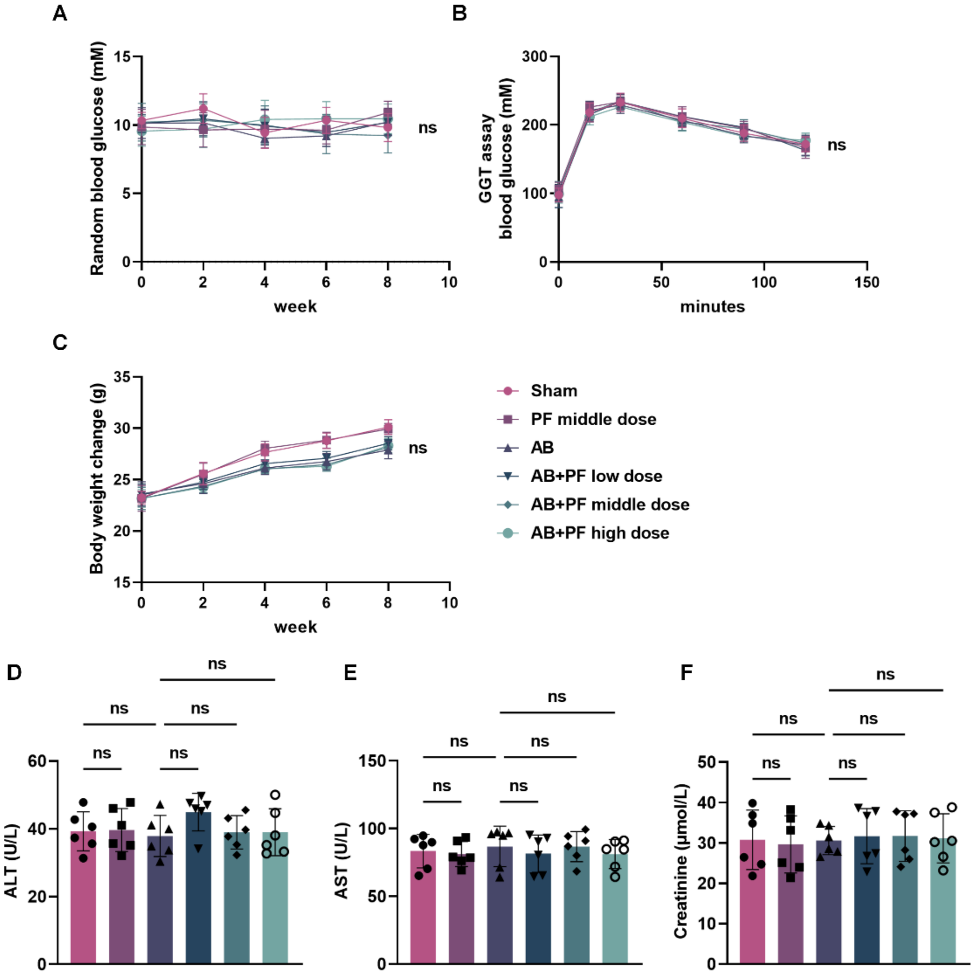

Figure S1 Effects of different doses of PF on body weight, blood glucose, liver and kidney function in mice 8 weeks after AB surgery.

A. Effects on fasting blood glucose in mice treated with different doses of PF every two weeks after sham or AB surgery (n=6). **B.** The results of the GTT after 8 weeks of treatment with different doses of PF (n=6). **C.** Effect of different doses of PF on body weight of mice after sham or AB surgery (n=6). **D-E.** Detection of the liver function indicators ALT and AST after 8 weeks of treatment with different doses of PF (n=6). **F.** Detection of the kidney function indicators creatinine after 8 weeks of treatment with different doses of PF (n=6). All data are presented as mean ± SD. Statistical analysis was performed using one-way ANOVA followed by *post hoc* Tukey test or unpaired *t*-test. *ns* means no statistical significance, $*P < 0.05$, $**P < 0.01$, $***P < 0.001$, $****P < 0.0001$. GTT, glucose tolerance test; ALT, liver enzymes alanine aminotransferase; AST, aspartate aminotransferase.

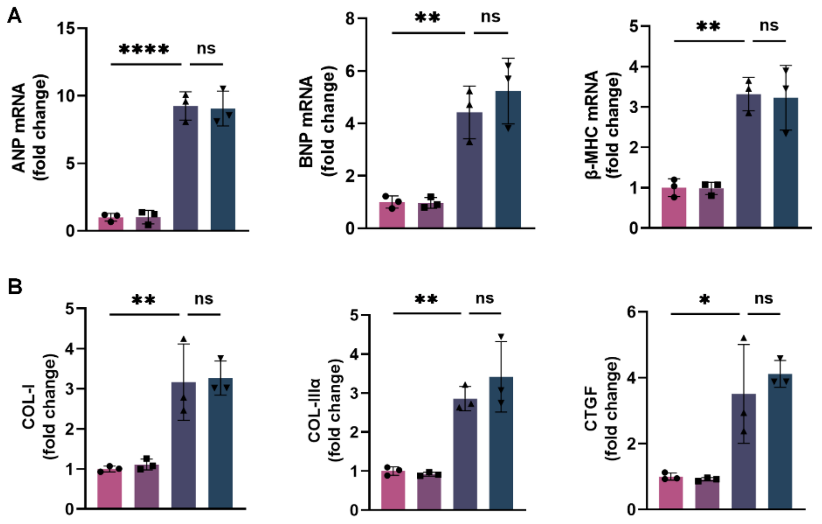

Figure S2 PF is unable to reverse pathological cardiac remodeling in AMPKα2$^{-/-}$ mice.

A. The mRNA expression of hypertrophic markers ANP, BNP and β-MHC in wild-type mice and AMPKα2$^{-/-}$ mice (*n*=3). **B.** The mRNA expression of myocardial fibrosis markers COL-I, COL-IIIα, CTGF (*n*=3). All data are presented as mean ± SD. Statistical analysis was performed using one-way ANOVA followed by *post hoc* Tukey test or unpaired *t*-test. *ns* means no statistical significance, ***$P < 0.001$, ****$P < 0.0001$.

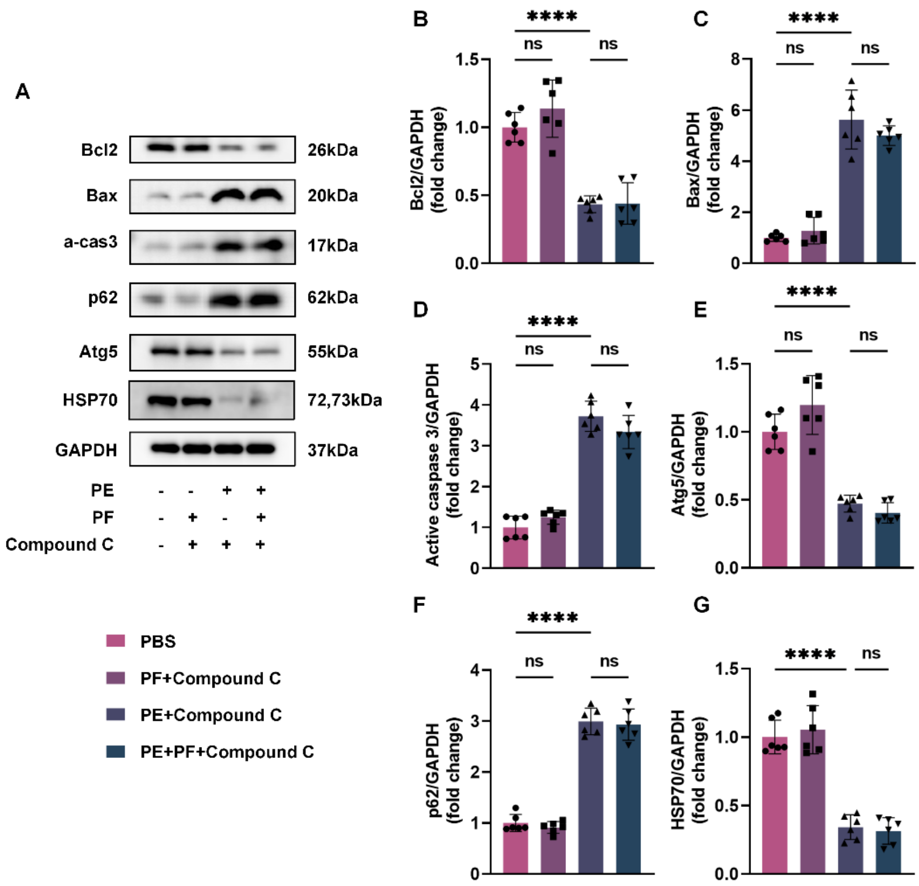

Figure S3 AMPKα inhibitors inhibit the protective effect of PF on cardiac remodeling.

A. The protein expression levels of Bcl-2, Bax, active-caspase3, p62, Atg5 and HSP70 in NRCMs treated with PF (n=6). **B-H.** Quantitative analysis of western blot data. All data are presented as mean ± SD. Statistical analysis was performed using one-way ANOVA followed by *post hoc* Tukey test or unpaired t-test. *ns* means no statistical significance, *$P < 0.05$, **$P < 0.01$, ***$P < 0.001$, ****$P < 0.0001$.
