# Supplementary material for: Danuglipron Ameliorates Pressure Overload‐Induced Cardiac Remodelling Through the AMPK Pathway
Source: J Cell Mol Med. 2025 Mar 11;29(5):e70488. doi: 10.1111/jcmm.70488 (PMC11897056; doi:10.1111/jcmm.70488)
Supplement: Supplementary file 2 — Table S1 [file JCMM-29-e70488-s002.docx]

**Supplemental material of Danuglipron ameliorates pressure overload-induced** **cardiac remodeling through the AMPK pathway**

**Author** Pan Wang^1,2#^, Zhen Guo^3,4#^, Chun-Yan Kong^1,2^, Yu-Lan Ma^1,2^, Ming-Yu Wang^1,2^, Xin-Ru Zhang^1,2^, Zheng Yang^1,2*^

**^*^Corresponding author:** Zheng Yang, Email: dr_yangzheng@whu.edu.cn

**Author's affiliation**

^1^ Department of Cardiology, Renmin Hospital of Wuhan University, Wuhan 430060, RP China

^2^ Hubei Key Laboratory of Metabolic and Chronic Diseases, Wuhan 430060, RP China

^3^ Department of Cardiology, Zhongnan Hospital of Wuhan University, Wuhan, 430062, China;

^4^ Institute of Myocardial Injury and Repair, Wuhan University, Wuhan, 430062, China.

^#^ These authors contributed equally: Pan Wang, Zhen Guo.

**Figure S1 Effects of different doses of PF on body weight, blood glucose, liver and kidney function in mice 8 weeks after AB surgery.**

**A.** Effects on fasting blood glucose in mice treated with different doses of PF every two weeks after sham or AB surgery (*n*=6). **B.** The results of the GTT after 8 weeks of treatment with different doses of PF (*n*=6). **C.** Effect of different doses of PF on body weight of mice after sham or AB surgery (*n*=6). **D-E.** Detection of the liver function indicators ALT and AST after 8 weeks of treatment with different doses of PF (*n*=6). **F.** Detection of the kidney function indicators creatinine after 8 weeks of treatment with different doses of PF (*n*=6). All data are presented as mean±SD. Statistical analysis was performed using one-way ANOVA followed by *post hoc* Tukey test or unpaired *t*-test. *ns* means no statistical significance.

GTT, glucose tolerance test; ALT, liver enzymes alanine aminotransferase; AST, aspartate aminotransferase.

**Figure S2 PF is unable to reverse pathological cardiac remodeling in** $\boldsymbol{AMPK\alpha2}^{\mathbf{-/-}}$ **mice.**

**A.** The mRNA expression of hypertrophic markers ANP, BNP and β-MHC in wild-type mice and ${AMPK\alpha2}^{-/-}$ mice (*n*=3). **B.** The mRNA expression of myocardial fibrosis markers COL-I, COL-IIIα, CTGF (*n*=3). All data are presented as mean±SD. Statistical analysis was performed using one-way ANOVA followed by *post hoc* Tukey test or unpaired *t*-test. *ns* means no statistical significance, **P* < 0.05, ***P* < 0.01, *****P* < 0.0001.

**Figure S3 AMPKα inhibitors inhibit the protective effect of PF on cardiac remodeling.**

**A.** The protein expression levels of Bcl-2, Bax, active-caspase3, p62, Atg5 and HSP70 in NRCMs treated with PF (*n*=6). **B-H.** Quantitative analysis of western blot data. All data are presented as mean±SD. Statistical analysis was performed using one-way ANOVA followed by *post hoc* Tukey test or unpaired *t*-test. *ns* means no statistical significance, *****P* < 0.0001.

Table S1 Primers used for of RT-PCR.

| Gene | Species | Forward primer (5’→3’) | Reverse primer (3’→5’) |
| --- | --- | --- | --- |
| ANP | Mouse | ACCTGCTAGACCACCTGGAG | CCTTGGCTGTTATCTTCGGTACCGG |
| BNP | Mouse | GAGGTCACTCCTATCCTCTGG | GCCATTTCCTCCGACTTTTCTC |
| β-MHC | Mouse | CCGAGTCCCAGGTCAACAA | CTTCACGGGCACCCTTGGA |
| COL-I | Mouse | AGGCTTCAGTGGTTTGGATG | CACCAACAGCACCATCGTTA |
| COL-IIIα | Mouse | CCCAACCCAGAGATCCCATT | GAAGCACAGGAGCAGGTGTAGA |
| CTGF | Mouse | ACTATGATGCGAGCCAACTGC | TGTCCGGATGCACTTTTTGC |
| GAPDH | Mouse | ACT​CCA​CTC​ACG​GCA​AAT​TC | TCTCCATGGTGGTGAAGACA |
| ANP | Rat | AAAGCAAACTGAGGGCTCTGCTCG | TTCGGTACCGGAAGCTGTTGCA |
| BNP | Rat | ACAATCCACGATGCAGAAGCT | GGGCCTTGGTCCTTTGAGA |
| β-MHC | Rat | TCTGGACAGCTCCCCATTCT | CAAGGCTAACCTGGAGAAGATG |
| GAPDH | Rat | GACATGCCGCCTGGAGAAAC | AGCCCAGGATGCCCTTTAGT |
